# Are Loneliness and Fear of Missing Out Linked to Social Media Addiction? Insights From a Cross‐Sectional Study Among Young Adults in Saudi Arabia

**DOI:** 10.1002/brb3.71137

**Published:** 2025-12-17

**Authors:** Mohannad A. Alzain, Ahmed K. Shukri, Abdullah Ahmed Qahti, Abdulaziz Hassan Abdullah Alali, Mohammed R. Algethami, Najim Z. Alshahrani

**Affiliations:** ^1^ Department of Family Medicine, Faculty of Medicine King Abdulaziz University Jeddah Saudi Arabia; ^2^ Family Medicine and Chronic Diseases Research Unit, King Fahd Medical Research Center King Abdulaziz University Jeddah Saudi Arabia; ^3^ Department of Family and Community Medicine, Faculty of Medicine University of Jeddah Jeddah Saudi Arabia; ^4^ Prince Sultan Military College of Health Sciences Dhahran Saudi Arabia

**Keywords:** fear of missing out, loneliness, Saudi Arabia, social media addiction, young adults

## Abstract

**Objectives:**

Despite the increasing trend of social media (SM) use in Saudi Arabia, few studies have examined the prevalence of social media addiction (SMA) and its psychological correlates, including loneliness and fear of missing out (FoMO), particularly among young adults. The purposes of this investigation were to: (i) assess the prevalence of SMA among Saudi young adults and (ii) evaluate how loneliness and FoMO are correlated to SMA.

**Methods:**

Data for cross‐sectional study were obtained from students, representing young adults, enrolled in three public universities in Saudi Arabia. *Bergen Social Media Addiction Scale*, *Revised UCLA Loneliness Scale (ULS‐6)* and *Fear of Missing Out Scale* were utilized to assess SMA, loneliness and FoMO, respectively. Linear regression models were applied to explore the predictors of SMA.

**Results:**

A total of 978 young adults participated in this study (average age 22.6 years). Half of the participants (52.8%) were addicted to SM. According to the adjusted linear regression model (adjusted *R* ^2^ = 0.804), the mean SMA score increased by 0.90 and 0.28 units for every unit increase in loneliness (regression coefficient, *β* = 0.90, 95% confidence interval, 95% CI = 0.86–0.94, *p* < 0.001) and FoMO (*β* = 0.28, 95% CI = 0.17–0.49, p < 0.018) scores, respectively.

**Conclusions:**

Loneliness and FoMO were significant predictors of SMA. These findings can benefit policymakers in developing and implementing public health policies related to SM use and psychological well‐being in Saudi Arabia.

## Graphical abstract

Social media addiction affected 52.8% of young adults, with loneliness and fear of missing out emerging as strong independent predictors. These findings underscore the need for policies supporting healthier digital behavior and psychological well‐being in Saudi Arabia.

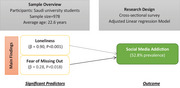

## 1 Introduction

With the substantial improvement in information and digital technologies, social networking sites have turned into a crucial aspect of today's world in the means of virtual communications. Over the last 20 years, the reach and popularity of social or online communication sites have expanded profoundly, particularly among the young people, who use these sites actively and frequently (Villanti et al. 2017). Although social media (SM) provides significant positive rewards, such as facilitating connections and enabling the rapid dissemination of information, growing concerns have emerged about its potential to foster addictive behaviors. Social media addiction (SMA), defined by uncontrollable and excessive use that disrupts daily functioning, mental health, and well‐being, has become a subject of increasing concern (Kuss and Griffiths 2017; O'Day and Heimberg 2021; Williams et al. 2024). Research suggests that psychological factors, including loneliness and fear of missing out (FoMO), may play critical roles in driving this addictive behavior (O'Day and Heimberg 2021; Wu et al. 2024; Zhu and Xiong 2022; Bakry et al. 2022), highlighting the need to better understand their interplay in the context of SM use.

In recent times, there has been growing concern over the rising rates of loneliness in modern society (Surkalim et al. 2022). Loneliness is a subjective experience of isolation, characterized by a perceived lack of meaningful social connections (social isolation) and a sense of emotional disconnection from others (emotional isolation). Emerging adulthood appears to be a particularly vulnerable period for experiencing loneliness, which is concerning given its links to negative health consequences (Kirwan et al. 2025). Some experts speculate that prolonged screen duration and SM use may be factors in this phenomenon; however, the relationship remains complex and context‐specific and requires further exploration (O'Day and Heimberg 2021; Twenge et al. 2018).

On the other hand, FoMO, a newer psychological construct, describes the persistent anxiety that others are enjoying fulfilling experiences without one's participation (Przybylski et al. 2013). FoMO is the feeling of tension, anxiety, and emptiness that occurs when someone feels cut off from what is happening in other people's lives. This includes events and situations outside their own life and what others are doing. It comes from a strong desire to be in the know at all times. This fear may lead people to obsessively check SM to keep track of what their peers are doing. Evidence indicates that those who experience a greater FoMO tend to engage more excessively with SM and exhibit addictive tendencies in their online behavior (Zhu and Xiong 2022; Servidio et al. 2024).

The Kingdom of Saudi Arabia presents a unique context for examining these phenomena. The country has witnessed a sharp rising mark in SM usage, with the number of user identities growing from 7.60 million in 2014 to a projected 35.33 million in 2024 (GMI Research Team 2023). This surge is particularly pronounced among young adults, who constitute a sizeable share of the population. Several studies have been done on SMA among diverse age groups (Alfaya et al. 2023; Al‐Abyadh 2025; Faqihi et al. 2024; Saud et al. 2019; Alsabaani et al. 2018; Halboub et al. 2016). For instance, a study of Saudi medical students found a 55.2% prevalence of SMA, with higher rates linked to male gender, depressive symptoms, and anxiety (Alfaya et al. 2023). Another recent university‐based study from Saudi Arabia identified a significant positive link between FoMO and SMA (Al‐Abyadh 2025). Despite the widespread use of SM, research exploring the prevalence of SMA and its psychological correlates like loneliness and FoMO remains limited among young Saudis.

### 1.1 Aims

The current study aims to address two primary objectives: (i) to report the prevalence of SMA among young adults in Saudi Arabia and (ii) to evaluate how loneliness and FoMO are correlated to SMA. By examining these relationships, this research seeks to expand existing knowledge about SMA. Furthermore, the findings of this study could inform the formulation and implementation of targeted interventions and awareness campaigns to mitigate the negative effects of SMA among Saudi young people. Given the cultural and social environment of Saudi Arabia, this study holds significant implications for public health and policy, emphasizing the need to balance the benefits of SM with its potential risks.

## 2 Methods and Materials

### 2.1 Survey Design

The design type of this survey was cross‐sectional. This investigation period was between January and June 2024 (approximately 6 months).

### 2.2 Participants and Procedure

Data were collected from both undergraduate and postgraduate students studying in the University of Jeddah (UOJ), King Faisal University (KFU), and King Khalid University (KKU), Saudi Arabia. The study received ethical approval [ECM#2023‐803] from the Institutional Research Ethics Committee of KKU. Data were collected using a structured, internet‐based questionnaire created with Google Forms. The survey link was distributed to students at the selected institutions via their institutional email addresses. The email invitation contained a summary of study objectives and ethics. The survey link was open for up to 1 month after the invitation.

### 2.3 Sample Size Calculation

We employed Cochran's formula to ascertain a statistically acceptable number of samples for this survey. The formula is $n=\frac{z^{2}\times p\times q}{d^{2}}$. Here, *n* is the sample size, *Z* is the precision level at 95%, *p* is the approximate percentage of a characteristic or an attribute that exists in the population, *q* = 1 − *p* and *d* = allowable error margin. Our estimation was based on the following assumptions: (i) *p* = 0.552 (as 55.2% prevalence of SMA among Saudi medical students) (Alfaya et al. 2023), (ii) *Z* = 1.96, and *d* = 0.05. Thus, by entering the numbers into the formula above, the calculation indicated that at least 380 participants was required.

### 2.4 Assessment of Outcome Variable

To assess SMA among young adults, we used *Bergen Social Media Addiction Scale (BSMAS)* (Andreassen et al. 2016). This scale assesses SMA by looking at the symptoms and negative consequences that come with problematic use over the past 12 months. The BSMAS has six items and five‐point Likert response options. The response choices vary from “very rarely” (one point) to “very often” (five points). The final score (range: 6–30) was calculated by summing up an individual's scores for each item. Elevated scores on the BSMAS correspond to increased level of SMA (SMA score was used as dependent variable). A strict polythetic classification approach was employed to assess the prevalence of SMA (Cheng et al. 2021). In this analysis, the BSMAS showed an adequate internal consistency (Cronbach's alpha = 0.76).

### 2.5 Assessment of Main Independent Variables

Loneliness was screened using the brief‐form of the *Revised UCLA Loneliness Scale* (ULS‐6) (Hussien 2022; Alheneidi et al. 2021). This six‐item scale offers a more concise alternative to the original 20‐item version (Russell et al. 1978). Responses were recorded on a Likert‐type scale from never (one) to often (four), with elevated values denoting greater loneliness. Previous research validated the ULS‐6's reliability within the Saudi population (Hussien 2022). This scale had acceptable reliability for this study (Cronbach's alpha: 0.71).

The *Fear of Missing Out Scale* (FoMOS), developed by Przybylski et al. (2013), was employed to assess participants’ levels of FoMO. The scale comprises 10 items, each rated on a five‐point Likert scale ranging from 1 (Not at all true for me) to 5 (Extremely true for me). The aggregate score ranges from 10 to 50, with higher points representing higher levels of FoMO. The FoMOS demonstrated good internal consistency, achieving a Cronbach's alpha of 0.79.

### 2.6 Other Covariates

Moreover, data on participants’ age, sex, education level, daily internet use duration, sleep status, physical activity, and smoking status were collected and included as covariates.

### 2.7 Data Analysis

Analysis was performed by STATA (BE version 17.0), and the significance level was specified at *p* < 0.05. Frequencies and percentages (categorical variables) and mean and standard deviation (continuous measures) were calculated. Pearson correlation was applied to observe the correlation between two continuous variables, and the results were depicted by simple scattered plots.

The scales for loneliness, FoMO, and SMA were treated as distinct theoretical constructs. To test this empirically and ensure the robustness of the regression model, the variance inflation factor (VIF) was calculated to assess multicollinearity among the independent variables. The results (mean VIF = 2.23, minimum VIF = 1.28, maximum VIF = 3.47) confirmed that multicollinearity does not pose a significant problem for the interpretation of the regression coefficients.

Unadjusted and adjusted linear regression models were applied to explore how loneliness and FoMO predict SMA. In these models, the scales were not combined but were entered as distinct independent variables to assess their individual contributions. Several demographic and other factors such as participants’ age, sex, education level, smoking status, sleeping status, regular physical exercise, and duration of daily internet use were incorporated in the adjusted linear regression model to observe the adjusted estimated effect of loneliness and FoMO on SMA. The assumptions of the adjusted linear regression model were tested and the model was fitted statistically. The normality assumption was tested using the Shapiro–Wilk test on the model residuals (*W* = 0.894, *p* = 0.1874). The non‐significant result indicates no substantial departure from normality, supporting this key regression assumption. Moreover, the Breusch–Pagan test indicated no evidence of heteroscedasticity (*χ* ^2^(1) = 0.83, *p* = 0.351). The results of linear regression analysis were presented as regression coefficient (*β*) and 95% confidence interval (CI).

## 3 Results

### 3.1 Participants’ Characteristics

A total of 978 young adults incorporated in the analysis. The study participants’ mean age was 22.6 years. Males made up more than half (55.7%) of the participants. A large share of the participants (77.6%) were studying in undergraduate level. Half of the participants used internet 7–9 h daily (49.9%) and slept less than normal hours (50.0%). Three‐quarters of the participants (77.9%) did not regularly engage in physical exercise. Twelve percent of the participants had smoking habits (Table 1).

**TABLE 1:** Study participants’ background information (*n* = 978).

Variables	Frequency (%)	Mean (SD)
**Age (in years)**		22.86 (1.92)
		Minimum age = 19
		Maximum age = 27
**Sex**		
Male	545 (55.7)	
Female	433 (44.3)	
**Education level**		
Undergraduate	759 (77.6)	
Post‐graduate	219 (22.4)	
**Duration of daily internet use**		
≤3 h	153 (15.6)	
4–6 h	337 (34.5)	
7–9 h	488 (49.9)	
**Sleeping status**		
Less than normal	489 (50.0)	
Normal	345 (36.3)	
More than normal	144 (14.7)	
**Regular physical exercise**		
Yes	216 (22.1)	
No	762 (77.9)	
**Smoking status**		
Yes	117 (12.0)	
No	861 (88.0)	
**ULS‐6 score (loneliness)**		15.85 (4.47)
		Minimum score = 5
		Maximum score = 24
**FoMOS score (fear of missing out)**		22.71 (7.36)
		Minimum score = 10
		Maximum score = 48

Abbreviation: FoMOS, *Fear of Missing Out Scale*.

### 3.2 SMA: Prevalence and Correlation With ULS‐6 and FoMOS Scores

**FIGURE 1:**
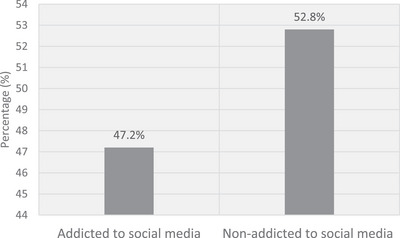
The proportion of study participants were addicted to social media (*n* = 978).

**FIGURE 2:**
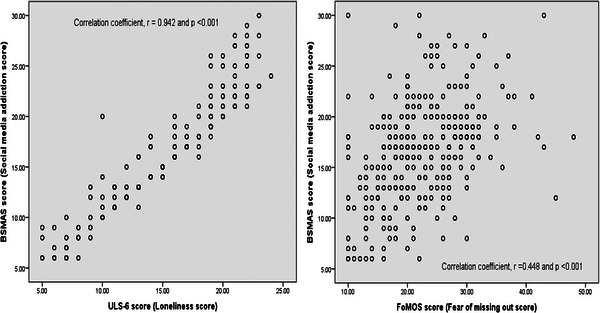
The correlation between social media addiction, loneliness, and fear of missing out is demonstrated by simple scatter plots (*n* = 978).

Approximately 53% (52.8%) of the participants were addicted to SM (Figure 1). The mean scores for the BSMAS, ULS‐6, and FoMOS were 15.90 (5.16), 15.85 (4.47), and 22.71 (7.36), respectively. As depicted in Figure 2 (left figure), a strong positive correlation was observed between participants’ SMA and loneliness (*r* = 0.942, p < 0.001). There was also a moderately positive link between SMA and FoMO (*r* = 0.448, p < 0.001) (Figure 2: right side).

Table 2 shows how loneliness and FoMO predict SMA by unadjusted and adjusted linear regression models. According to the unadjusted linear regression estimate, SMA scores and loneliness scores were positively correlated (*β* = 1.07, 95% CI = 1.04–1.09, p < 0.001). After controlling for demographic and other factors (e.g., participants’ age, sex, education level, smoking status, sleeping status, regular physical exercise, and duration of daily internet use), the adjusted linear regression model revealed a statistically significant positive correlation between loneliness scores and SMA scores (*β* = 0.90, 95% CI = 0.86–0.94, p < 0.001). On the basis of this model, the mean SMA score increased by 0.90 units for every unit increase in loneliness (Table 2).

**TABLE 2:** Linear regression analysis reveals that loneliness and fear of missing out are associated with social media addiction among young adults in Saudi Arabia.

Statistics	Predictor variables	
	Loneliness score	Fear of missing out score
Model I: Unadjusted estimate		
Regression coefficient	1.07	0.31
95% confidence interval	1.04–1.09	0.27–0.55
p value	<0.001	<0.001
Model II: Adjusted estimate		
Regression coefficient	0.90	0.28
95% confidence interval	0.86–0.94	0.17–0.49
p value	<0.001	0.018

*Note*: The model II was adjusted for participants’ age, sex, education level, smoking status, sleeping status, regular physical exercise, and duration of daily internet use. The adjusted model was statistically significant (*p* = 0.000). The adjusted *R* ^2^ for the model II was 0.804.

Moreover, FoMO scores were found to be positively associated with SMA scores in the unadjusted linear regression model (*β* = 0.31, 95% CI = 0.27–0.55, p < 0.001). After adjusting for demographic and other factors, the adjusted linear regression model demonstrated a statistically significant positive association between FoMO scores and SMA scores (*β* = 0.28, 95% CI = 0.17–0.49, p < 0.018). The finding shows that the participants’ mean SMA score increased by 0.28 units when their FoMO score increased by 1 unit (Table 2).

## 4 Discussion

There were two key findings in this research: (i) SMA was observed in approximately 53% of Saudi young adults who participated in this study. (ii) Loneliness and FoMO were significant predictors of SMA among study participants. These findings add factual information to the concurrent literature and can benefit policymakers in developing and implementing public health regulations pertaining to SM use and psychological well‐being in Saudi Arabia.

The observed rate of SMA in this investigation was consistent with prior studies conducted among medical (55.2% prevalence) and university students (50.1% had moderate SMA) in Saudi Arabia (Alfaya et al. 2023; Saud et al. 2019). However, another study in Saudi Arabia reported higher rates of SMA (74%) compared to the current investigation (Bakry et al. 2022). This study's observed rate is even higher than the reported global prevalence of SMA (18.4% pooled prevalence) among university students (included 51 studies, sample size: 35,520 students) (Salari et al. 2023). Although differences in rates of SMA vary due to sample sizes, measurement scales, and cut‐off points, the increasing trend of SMA among young adults in Saudi Arabia is extremely worrying. To curb the rising trend of SMA in Saudi Arabia, policymakers should implement public awareness and education programs. These programs should include: (i) launching nationwide awareness campaigns on the consequences of over use of SM. (ii) Educating young adults about digital well‐being through schools, universities, and workplaces. (iii) Promoting responsible SM use by encouraging mindful scrolling and setting time limits.

The current study revealed that loneliness is associated with SMA, which aligns with several studies (O'Day and Heimberg 2021; Wu et al. 2024; Bakry et al. 2022; Reissmann et al. 2018). A systematic review analyzing 52 articles concluded that loneliness is a risk factor for problematic SM use (O'Day and Heimberg 2021). Kross et al. (2013) revealed that experiencing loneliness at a particular time predicted an increase in Facebook use over time among young adults. A study in Turkey reported that university students’ SM scores and loneliness scores are positively correlated (Uyaroğlu et al. 2022). Furthermore, there is concrete literature of a bidirectional causal relationship (i.e., positive influence on each other) between loneliness and problematic SM use (Wu et al. 2024). However, evidence also confirmed that greater problematic use of SM is connected with higher loneliness (Williams et al. 2024; Youssef et al. 2020). Given the mixed evidence on the directional relationship between loneliness and SMA, further longitudinal and experimental studies are recommended to explore these relationships in Saudi Arabia.

A possible rationale for justifying how loneliness can contribute to SMA among young adults in Saudi Arabia broadly involves two factors: (i) SM as an escape mechanism for isolation, and (ii) cultural and societal factors. First, loneliness often drives individuals to seek solace in online platforms, where they can engage in virtual interactions (Primack et al. 2017). SM provides an accessible avenue for young adults to fulfill their need for social connection, albeit in a superficial or transient manner (Twenge et al. 2018). Thus, excessive reliance on SM as a coping technique for loneliness can lead to addictive behaviors, as individuals may become dependent on the instant gratification and validation these platforms provide (Andreassen et al. 2017). Additionally, feelings of loneliness may lead people to browse through SM feeds passively (i.e., loneliness also predicts passive SM use) (Aalbers et al. 2019). Finally, in the context of Saudi Arabia, cultural and societal factors may exacerbate this relationship. For instance, cultural norms, including gender segregation and conservative social practices, may limit in‐person interactions, particularly between genders, pushing young adults to rely more heavily on SM for communication and social engagement.

This study also revealed that FoMO was positively correlated with SMA, indicating that participants with FoMO were more likely to be addicted to SM. This finding is supported by several existing studies (Zhu and Xiong 2022; Servidio et al. 2024; Al‐Abyadh 2025; Zhang et al. 2021; Varchetta et al. 2020; Sultan 2021). Individuals experiencing FoMO, compulsive need to maintain continuous social connection, tend to enhance their self‐presentation behavior on SM, which subsequently turn into SMA (Zhu and Xiong 2022). Research indicates that increased FoMO is associated with an adverse impact of SM on daily activities and work productivity (Rozgonjuk et al. 2020).

By establishing the bivariate relationships, this study supports the necessary groundwork for future research to test these more complex, mediated pathways. Existing research suggests underlying mediating mechanisms, where FoMO acts as a mediator. For example, research with older Chinese adults found that loneliness can lead to problematic use through a chain of sensation seeking and FoMO (Cui et al. 2024). It suggests that similar cognitive and emotional processes, likely involving FoMO, drive the dynamics seen in Saudi young adults. An important direction for future research is to longitudinally test these mediation models to confirm the specific psychological drivers of SMA in this population. Identifying these mediating roles would allow for a more focused approach to interventions in these populations.

### 4.1 Implications of Research Findings

The positive association between loneliness and SMA highlights the need for interventions that address the causes of loneliness while promoting and encouraging healthier digital behaviors. For example, fostering opportunities for in‐person social interactions and community engagement could help mitigate the reliance on SM as a primary source of connection. Additionally, awareness campaigns about the demerits of SMA and promoting digital literacy could empower young adults to use these platforms more mindfully. Provide accessible counseling services for young adults struggling with SMA and FoMO‐related stress. Train mental health professionals to address digital addiction as part of routine psychological support services.

### 4.2 Strengths and Limitations

The strengths of this study include the following:

- A relatively large sample size of young adults from Saudi Arabia.
- The use of standardized, psychometrically validated instruments. Specifically, the BSMAS, FoMOS, and ULS‐6 were all previously validated and used in the Saudi context (Hussien 2022; Abiddine et al. 2024; Al‐Menayes 2016), enhancing the cultural appropriateness and reliability of our data.
- The use of robust statistical analyses (adjusted linear regression) to control for potential confounders.

The study had some notable limitations, which include the following:

- The ability to establish causal inference is hindered because of cross‐sectional study design. Future research could employ longitudinal or experimental designs to better explore causality.
- This study covered three universities which limits the sample representativeness. A broader, more diverse sample in future work would help confirm the external validity of these findings.
- The participant recruitment procedures restricted the external validity of this study.
- This study focused specifically on loneliness and FoMO and therefore did not explore other potential contributing factors to SMA, such as personality traits or underlying mental health conditions. Future research should incorporate a broader range of psychological and social variables to develop a more comprehensive model of addiction.
- Our findings are unable to specify which SM platforms are most associated with addiction due to the lack of platform‐specific data. Consequently, future studies should classify and examine different SM types to determine which digital environments pose the greatest risk for young adults.
- Self‐reporting biases may have been present among participants.

## 5 Conclusion

Half of the participating young adults were addicted to SM. Loneliness and FoMO were two significant predictors of SMA. Further longitudinal studies are required to infer the causal association between psychological factors (such as loneliness and FoMO) and SMA.

## Author Contributions

**Mohannad A. Alzain**: methodology, investigation, funding, resources: writing – original draft. **Ahmed K. Shukri**: investigation, data curation, writing – review and editing. **Abdullah Ahmed Qahti**: investigation, data curation, writing – review and editing. **Abdulaziz Hassan Abdullah Alali**: investigation, data curation, writing – review and editing. **Mohammed R. Algethami**: data curation, writing – review and editing. **Najim Z. Alshahrani**: conceptualization, methodology, formal analysis, writing – original draft, writing – review and editing, supervision, project administration. All authors contributed substantially to the work, reviewed the manuscript critically, and approved the final version. Mohammed R. Algethami and Najim Z. Alshahrani jointly conducted the data analysis and co‐wrote the manuscript.

## Conflicts of Interest

The authors declare no conflicts of interest.

## Data Availability

The data that support the findings of this study are available from the corresponding author upon reasonable request.

## References

[brb371137-bib-0001] Aalbers, G. , R. J. McNally , A. Heeren , S. De Wit , and E. I. Fried . 2019. “Social Media and Depression Symptoms: A Network Perspective.” Journal of Experimental Psychology General 148, no. 8: 1454–1462. 30507215 10.1037/xge0000528

[brb371137-bib-0002] Abiddine, F. Z. , M. A. Aljaberi , A. Alduais , C. Y. Lin , Z. Vally , and M. D Griffiths . 2024. “The Psychometric Properties of the Arabic Bergen Social Media Addiction Scale.” International Journal of Mental Health and Addiction 23: 3395–3415.

[brb371137-bib-0003] Al‐Abyadh, M. H. A. 2025. “The Fear of Missing Out and Social Media Addiction: A Cross‐Sectional and Quasi‐Experimental Approach.” Heliyon 11, no. 3: e41958. 39975830 10.1016/j.heliyon.2025.e41958 PMC11835568

[brb371137-bib-0004] Alfaya, M. A. , N. S. Abdullah , N. Z. Alshahrani , et al. 2023. “Prevalence and Determinants of Social Media Addiction Among Medical Students in a Selected University in Saudi Arabia: A Cross‐Sectional Study.” In Healthcare. MDPI. 10.3390/healthcare11101370 PMC10217812 37239655

[brb371137-bib-0005] Alheneidi, H. , L. AlSumait , D. AlSumait , and A. P. Smith . 2021. “Loneliness and Problematic Internet Use During COVID‐19 Lock‐Down.” Behavioral Sciences 11, no. 1: 5. 33418914 10.3390/bs11010005 PMC7825032

[brb371137-bib-0006] Al‐Menayes, J. 2016. “The Fear of Missing Out Scale: Validation of the Arabic Version and Correlation With Social Media Addiction.” International Journal of Applied Psychology 6, no. 2: 41–46.

[brb371137-bib-0007] Alsabaani, A. , A. A. Alshahrani , A. S. Abukaftah , and S. F. Abdullah . 2018. “Association Between Over‐Use of Social Media and Depression Among Medical Students, King Khalid University, Kingdom of Saudi Arabia.” Egyptian Journal of Hospital Medicine 70, no. 8: 1305–1311.

[brb371137-bib-0008] Andreassen, C. S. , J. Billieux , M. D. Griffiths , et al. 2016. “The Relationship Between Addictive Use of Social Media and Video Games and Symptoms of Psychiatric Disorders: A Large‐Scale Cross‐Sectional Study.” Psychology of Addictive Behaviors 30, no. 2: 252–262. 26999354 10.1037/adb0000160

[brb371137-bib-0009] Andreassen, C. S. , S. Pallesen , and M. D. Griffiths . 2017. “The Relationship Between Addictive Use of Social Media, Narcissism, and Self‐Esteem: Findings From a Large National Survey.” Addictive Behaviors 64: 287–293. 27072491 10.1016/j.addbeh.2016.03.006

[brb371137-bib-0010] Bakry, H. , A. A. Almater , D. M. Alslami , et al. 2022. “Social Media Usage and Loneliness Among Princess Nourah University Medical Students.” Middle East Current Psychiatry 29, no. 1: 50.

[brb371137-bib-0011] Cheng, C. , Y. Lau , L. Chan , and J. W. Luk . 2021. “Prevalence of Social Media Addiction Across 32 Nations: Meta‐Analysis With Subgroup Analysis of Classification Schemes and Cultural Values.” Addictive Behaviors 117: 106845. 33550200 10.1016/j.addbeh.2021.106845

[brb371137-bib-0012] Cui, S. , J. Jiang , and L. Mu . 2024. “The Relationship Between Loneliness and the Overuse of WeChat Among Chinese Elderly: The Chain Mediation Role of Sensation Seeking and Fear of Missing Out.” Psychology Research and Behavior Management 17: 3067–3081. 39220632 10.2147/PRBM.S467221 PMC11363962

[brb371137-bib-0013] Faqihi, F. A. , R. A. Qutob , R. H. M. Subh , et al. 2024. “Examining the Effects of Social Media on Mental Health Among Adolescents in Saudi Arabia.” Cureus 16, no. 1: e53261. 38435934 10.7759/cureus.53261 PMC10904877

[brb371137-bib-0014] GMI Research Team . 2023. “Saudi Arabia Social Media Statistics 2024.” Accessed March 6, 2023. https://www.globalmediainsight.com/blog/saudi‐arabia‐social‐media‐statistics/.

[brb371137-bib-0015] Halboub, E. , F. Othathi , F. Mutawwam , S. Madkhali , D. Somaili , and N. Alahmar . 2016. “Effect of Social Networking on Academic Achievement of Dental Students, Jazan University, Saudi Arabia.” Eastern Mediterranean Health Journal 22, no. 12: 865–871. 10.26719/2016.22.12.865 28181661

[brb371137-bib-0016] Hussien, R. M. 2022. “The Association Between Nomophobia and Loneliness Among the General Population in the Kingdom of Saudi Arabia.” Middle East Current Psychiatry 29, no. 1: 68.

[brb371137-bib-0017] Kirwan, E. M. , A. Burns , P. S. O'Súilleabháin , et al. 2025. “Loneliness in Emerging Adulthood: A Scoping Review.” Adolescent Research Review 10: 47–67.

[brb371137-bib-0018] Kross, E. , P. Verduyn , E. Demiralp , et al. 2013. “Facebook Use Predicts Declines in Subjective Well‐Being in Young Adults.” PLoS ONE 8, no. 8: e69841. 23967061 10.1371/journal.pone.0069841 PMC3743827

[brb371137-bib-0019] Kuss, D. J. , and M. D. Griffiths . 2017. “Social Networking Sites and Addiction: Ten Lessons Learned.” International Journal of Environmental Research and Public Health 14, no. 3: 311. 28304359 10.3390/ijerph14030311 PMC5369147

[brb371137-bib-0020] O'Day, E. B. , and R. G. Heimberg . 2021. “Social Media Use, Social Anxiety, and Loneliness: A Systematic Review.” Computers in Human Behavior Reports 3: 100070.

[brb371137-bib-0021] Primack, B. A. , A. Shensa , J. E. Sidani , et al. 2017. “Social Media Use and Perceived Social Isolation Among Young Adults in the US.” American Journal of Preventive Medicine 53, no. 1: 1–8. 28279545 10.1016/j.amepre.2017.01.010 PMC5722463

[brb371137-bib-0022] Przybylski, A. K. , K. Murayama , C. R. DeHaan , and V. Gladwell . 2013. “Motivational, Emotional, and Behavioral Correlates of Fear of Missing Out.” Computers in Human Behavior 29, no. 4: 1841–1848.

[brb371137-bib-0023] Reissmann, A. , J. Hauser , E. Stollberg , I. Kaunzinger , and K. W. Lange . 2018. “The Role of Loneliness in Emerging Adults' Everyday Use of Facebook – An Experience Sampling Approach.” Computers in Human Behavior 88: 47–60.

[brb371137-bib-0024] Rozgonjuk, D. , C. Sindermann , J. D. Elhai , and C. Montag . 2020. “Fear of Missing Out (FoMO) and Social Media's Impact on Daily‐Life and Productivity at Work: Do WhatsApp, Facebook, Instagram, and Snapchat Use Disorders Mediate That Association?” Addictive Behaviors 110: 106487. 32674020 10.1016/j.addbeh.2020.106487

[brb371137-bib-0025] Russell, D. , L. A. Peplau , and M. L. Ferguson . 1978. “Developing a Measure of Loneliness.” Journal of Personality Assessment 42, no. 3: 290–294. 660402 10.1207/s15327752jpa4203_11

[brb371137-bib-0026] Salari, N. , H. Zarei , A. Hosseinian‐Far , S. Rasoulpoor , S. Shohaimi , and M. Mohammadi . 2023. “The Global Prevalence of Social Media Addiction Among University Students: A Systematic Review and Meta‐Analysis.” Journal of Public Health (Bangkok) 33: 223–236.

[brb371137-bib-0027] Saud, D. F. A. , S. A. Alhaddab , S. M. Alhajri , N. S. Alharbi , S. A. Aljohar , and E. M. Mortada . 2019. “The Association Between Body Image, Body Mass Index and Social Media Addiction Among Female Students at a Saudi Arabia Public University.” Malaysian Journal of Medicine and Health Sciences 15, no. 1: 16–22.

[brb371137-bib-0028] Servidio, R. , P. Soraci , M. D. Griffiths , S. Boca , and Z. Demetrovics . 2024. “Fear of Missing Out and Problematic Social Media Use: A Serial Mediation Model of Social Comparison and Self‐Esteem.” Addictive Behaviors Reports 19: 100536. 38495391 10.1016/j.abrep.2024.100536 PMC10943642

[brb371137-bib-0029] Sultan, A. J. 2021. “Fear of Missing Out and Self‐Disclosure on Social Media: The Paradox of Tie Strength and Social Media Addiction Among Young Users.” Young Consumers 22, no. 4: 555–577.

[brb371137-bib-0030] Surkalim, D. L. , M. Luo , R. Eres , et al. 2022. “The Prevalence of Loneliness Across 113 Countries: Systematic Review and Meta‐Analysis.” BMJ 376: e067068. 35140066 10.1136/bmj-2021-067068 PMC8826180

[brb371137-bib-0031] Twenge, J. M. , T. E. Joiner , M. L. Rogers , and G. N. Martin . 2018. “Increases in Depressive Symptoms, Suicide‐Related Outcomes, and Suicide Rates Among US Adolescents After 2010 and Links to Increased New Media Screen Time.” Clinical Psychological Science 6, no. 1: 3–17.

[brb371137-bib-0032] Uyaroğlu, A. K. , E. Ergin , A. S. Tosun , and Ö. Erdem . 2022. “A Cross‐Sectional Study of Social Media Addiction and Social and Emotional Loneliness in University Students in Turkey.” Perspectives in Psychiatric Care 58, no. 4: 2263–2271. 35152424 10.1111/ppc.13056

[brb371137-bib-0033] Varchetta, M. , A. Fraschetti , E. Mari , and A. M. Giannini . 2020. “Social Media Addiction, Fear of Missing out (FoMO) and Online Vulnerability in University Students.” Revista Digital de Investigación en Docencia Universitaria 14, no. 1: e1187.

[brb371137-bib-0034] Villanti, A. C. , A. L. Johnson , V. Ilakkuvan , M. A. Jacobs , A. L. Graham , and J. M. Rath . 2017. “Social Media Use and Access to Digital Technology in US Young Adults in 2016.” Journal of Medical Internet Research [Electronic Resource] 19, no. 6: e196. 28592394 10.2196/jmir.7303 PMC5480010

[brb371137-bib-0035] Williams, M. , K. M. Lewin , and D. Meshi . 2024. “Problematic Use of Five Different Social Networking Sites Is Associated With Depressive Symptoms and Loneliness.” Current Psychology 43: 20891–20898.

[brb371137-bib-0036] Wu, P. , R. Feng , and J. Zhang . 2024. “The Relationship Between Loneliness and Problematic Social Media Usage in Chinese University Students: A Longitudinal Study.” BMC Psychology 12, no. 1: 13. 38178215 10.1186/s40359-023-01498-4 PMC10765645

[brb371137-bib-0037] Youssef, L. , R. Hallit , N. Kheir , S. Obeid , and S. Hallit . 2020. “Social Media Use Disorder and Loneliness: Any Association Between the Two? Results of a Cross‐Sectional Study Among Lebanese Adults.” BMC Psychology 8: 56. 32487222 10.1186/s40359-020-00421-5 PMC7268264

[brb371137-bib-0038] Zhang, Y. , Y. Chen , J. Jin , and G. Yu . 2021. “The Relationship Between Fear of Missing out and Social Media Addiction: A Cross‐Lagged Analysis.” Chinese Journal of Clinical Psychology 5: 1082–1085.

[brb371137-bib-0039] Zhu, X. , and Z. Xiong . 2022. “Exploring Association Between Social Media Addiction, Fear of Missing Out, and Self‐Presentation Online Among University Students: A Cross‐Sectional Study.” Frontiers in Psychiatry 13: 896762. 35633794 10.3389/fpsyt.2022.896762 PMC9136033

